# Editorial: Cognitive stimulants: from caffeine to cannabinoids - current and future perspectives

**DOI:** 10.3389/fnbeh.2025.1547970

**Published:** 2025-01-23

**Authors:** Daniel Moreira-Silva, Marta C. Cunha-Rodrigues, Ana Elisa Speck, Pablo Pandolfo

**Affiliations:** ^1^Byrd Alzheimer's Center and Research Institute, University of South Florida, Tampa, FL, United States; ^2^Pharmacology and Psychobiology Department, Roberto Alcantara Gomes Biology Institute, State University of Rio de Janeiro, Rio de Janeiro, Brazil; ^3^Federal University of Santa Catarina, Florianopolis, Santa Catarina, Brazil; ^4^Fluminense Federal University, Niterói, Rio de Janeiro, Brazil

**Keywords:** coffee, memory, cannabis, cognition, cognitive enhancer

There is an emerging demand for cognitive enhancement in recent years, led by increasing pressure to maintain high mental performance in academic, professional, and personal spheres. Cognitive stimulants have gained popularity as faster-acting alternatives in comparison to traditional and more challenging-to-follow strategies like a balanced routine of exercise, nutrition, and sleep. This Research Topic comprehensively explores recent studies that uncover the intricate interactions among cognitive enhancers, neural substrates, and pharmacological side effects. Widely used substances such as caffeine and cannabinoids were emphasized while intriguing findings on novel compounds, such as Icariin, were also discussed. Importantly, the collection recognizes that non-pharmacological factors, such as environmental enrichment, play a pivotal role in cognitive enhancement, underscoring the need for an integrated approach to optimizing cognitive function.

## Caffeine: the everyday cognitive booster with a sharp edge

Caffeine is by far the most consumed cognitive stimulant worldwide, primarily due to its widespread availability in beverages like coffee, tea, and energy drinks. While caffeine is renowned for its ability to improve alertness and focus, there are growing concerns about its anxiogenic effects at higher doses. Consistent with previous data in the literature, Bao et al.'s study showed that moderate doses of caffeine (around 90 mg) may alleviate depressive symptoms. On the flip side, factors like sleep quality, education, and exercise may have influenced these outcomes, requiring further investigation. Liu et al. conducted a meta-analysis involving 546 healthy individuals across eight studies and found that caffeine intake, particularly in doses above 400 mg per day, significantly increased the risk of anxiety. This finding brings light to a concern of high epidemiological relevance, given caffeine's popularity, and calls for moderation in its use, especially for individuals with a predisposition to anxiety disorders. Picó-Pérez et al.'s fMRI analysis further differentiates caffeine's effects from the overall coffee-drinking experience. They revealed that habitual coffee intake boosts connectivity in the higher visual and right executive control networks but reduces it in the posterior default mode network and somatosensory/motor networks. Caffeine alone, however, only affects the posterior default mode, suggesting additional bioactive components contribute to cognitive modulation. Altogether, these findings underscore the nuanced balance between cognitive benefits and potential psychological downsides of caffeine consumption in humans. Additional studies examining the effects of a wider range of doses of caffeine in different physiological and pathological contexts are needed to elucidate the intricacies of the dose-response relationship.

## Cannabis and cannabinoids: dual roles in cognitive modulation

Similarly, cannabinoids, as plant-derived pharmaceutical agents, also exhibit a complex dose-response relationship in cognitive modulation, offering potential benefits but posing risks depending on dosage, individual health status, and context of use. Cannabinoids are chemical compounds found in plants such as *Cannabis sativa* and *Cannabis indica*, including tetrahydrocannabinol (THC), which is psychoactive, and cannabidiol (CBD), which is non-psychoactive and known for its therapeutic potential. Ognibene et al.'s study demonstrated that daily exposure to inhaled cannabis (containing 10.3% Δ9-THC) reduces brain sensitivity to Adderall, a drug commonly prescribed for narcolepsy and ADHD, particularly in dopaminergic pathways. Through detailed analysis using statistical heat maps and 3D reconstructions of 134 mouse brain regions, the study revealed that Adderall-induced activation patterns in reward and attention networks are suppressed in cannabis-exposed subjects. In the same direction, Beyer et al.'s article synthesized fMRI analysis on the brains of 534 individuals and noticed that the reward function is remarkably altered in cannabis users. Schouten et al.'s review on CBD highlights its therapeutic potential for neurological and psychiatric disorders, including epilepsy, Alzheimer's, and anxiety. CBD's ability to counteract the psychotic effects of THC illustrates the dual nature of cannabinoids. Altogether, these studies draw attention to the potential for cannabis to interfere with the effects of other cognitive stimulants and raise concerns about stimulant misuse.

## Novel cognitive enhancers and environmental influences: balancing therapeutic potential and possible caveats

The field of cognitive enhancement offers a spectrum of possibilities, from well-established stimulants like caffeine and methylphenidate to emerging natural compounds such as Icariin. Findings by Wang et al. reveal that Icariin, derived from the Epimedium plant, shows promising therapeutic potential by mitigating surgery-induced memory impairment, reducing hippocampal inflammation, and protecting against neuronal injury in elderly individuals with postoperative cognitive dysfunction (POCD).

However, cognitive function is not solely determined by pharmacological interventions. Research by Herrera-Isaza et al. demonstrates that environmental enrichment—which includes cognitive, sensory, and social stimulation—can alleviate emotional and cognitive dysfunction caused by methylphenidate. This underscores the importance of combining pharmacological treatments with supportive environments to achieve optimal cognitive outcomes.

The need for careful, multifactor evaluation of cognitive enhancers is further emphasized by Marques et al.'s review of therapies for hypoxic-ischemic encephalopathy (HIE). While substances like erythropoietin and melatonin present promising potential in preclinical models, their efficacy in humans remains unconfirmed in the context of cognitive dysfunction. This gap highlights the challenge of translating animal research into clinical practice and the importance of assessing both the benefits and risks of cognitive enhancers, particularly their long-term implications.

## Toward an integrated understanding of cognitive enhancement

The research presented in this collection illustrates the complexity of cognitive enhancement. From the cognitive-boosting potential of caffeine and methylphenidate to novel compounds like Icariin, these enhancers offer hope for improving cognitive function, especially for individuals under neuropathological conditions. Yet, studies on cannabinoids and environmental enrichment reveal that these substances do not act in isolation. Individual health status, dosage, and environmental factors all influence their effectiveness and safety.

This nuanced understanding is crucial for developing therapies that enhance cognition while protecting the brain from secondary psychological effects and avoiding unnecessary risks that could increase or exacerbate mental health disturbances. A multidimensional approach holds promise not only for individuals with cognitive impairments but also for those aiming to optimize their mental performance in a healthy, sustainable manner.

